# Superparamagnetic Iron Oxide Nanoparticles Carrying Chemotherapeutics Improve Drug Efficacy in Monolayer and Spheroid Cell Culture by Enabling Active Accumulation

**DOI:** 10.3390/nano10081577

**Published:** 2020-08-11

**Authors:** Khanh Nguyen, Bianca Nuß, Marina Mühlberger, Harald Unterweger, Ralf P. Friedrich, Christoph Alexiou, Christina Janko

**Affiliations:** 1 Department of Otorhinolaryngology, Head and Neck Surgery, Section of Experimental Oncology and Nanomedicine (SEON), Else Kröner-Fresenius-Stiftung Professorship, Universitätsklinikum Erlangen, 91054 Erlangen, Germany; khanh.nguyen@fau.de (K.N.); bianca.nuss@uk-erlangen.de (B.N.); harald.unterweger@uk-erlangen.de (H.U.); ralf.friedrich@uk-erlangen.de (R.P.F.); christoph.alexiou@uk-erlangen.de (C.A.); 2 Friedrich-Alexander-Universität Erlangen-Nürnberg (FAU), 91054 Erlangen, Germany

**Keywords:** tumor spheroids, superparamagnetic iron oxide nanoparticles, magnetic drug targeting, nanomedicine, chemotherapy, drug resistance, mitoxantrone

## Abstract

Cytotoxic and cytostatic chemotherapeutics act by attacking rapidly dividing tumor cells, predominantly affecting malignant tissue and to a certain degree preserving healthy cells. Nonetheless, severe side effects are caused as quickly proliferating healthy cells such as hematopoietic precursors and mucous membranes are impaired as well. This limits the administered dose and eventually allows tumor cells to escape treatment. In order to increase intratumoral drug concentration and simultaneously reduce systemic side effects, nanoparticles have come into focus as drug carriers. The functionalization of superparamagnetic iron oxide nanoparticles (SPIONs) with chemotherapeutics such as mitoxantrone (MTO) enables targeted drug transport by using magnetic forces. Here, we investigate SPIONs consisting of individual iron oxide cores of 10 nm in diameter and a total hydrodynamic diameter of 53 ± 0.8 nm as a transporting system for MTO. Comparing the killing efficacy in monolayer cell culture and multicellular tumor spheroids of HT-29 cells, we show that spheroids tolerate considerably higher doses of nanoparticle-loaded MTO. Therefore, dose predictions from conventional monolayer cell cultures are often misleading for in vivo applications. This was true for both soluble and nanoparticle-bound MTO. Using flow chambers mimicking in vivo blood flow, we furthermore demonstrate that SPIONs can magnetically accumulate MTO. We conclude that SPIONs can function as an effective delivery platform to increase local drug concentrations, thereby potentially overcoming chemotherapy resistance of cells.

## 1. Introduction

Alongside surgery, radiotherapy and immunotherapy, chemotherapy is one of the established strategies to treat cancer, alone or in combination with other techniques [1]. Cytotoxic and cytostatic chemotherapeutics of various classes induce deoxyribonucleic acid (DNA) damage in cells and cause cell cycle block to prevent cell division, eventually leading to apoptosis or senescence [2]. Conventional chemotherapy is known to act on quickly proliferating cells and thus entails cytotoxicity in all tissues regardless of malignancy or non-malignancy [3]. Due to their fast growth, hematopoietic precursors, hair follicle cells, and mucous membranes of the mouth, stomach and intestines are affected as well, causing a wide range of side effects which limit the therapeutic dose [4,5,6]. Problematically, systemically already toxic but insufficient doses may allow tumor cells to escape treatment and finally to develop drug resistance [7,8,9]. Also, there is evidence for the treatment with cytostatics to foster metastasis by triggering intravasation of tumor cells, even if the tumor size is decreased [10]. Several concepts have been proposed to achieve optimized drug transport in order to increase intratumoral drug concentration, to minimize systemic side effects or to overcome physiological barriers [11,12]. Many of these approaches involve local enrichment, e.g., by use of specific molecules, sequences or drug transporters to target a distinct area [13,14]. For instance, superparamagnetic iron oxide nanoparticles (SPIONs) can serve as carriers to accumulate drugs by use of magnetic forces, referred to as magnetic drug targeting (MDT) [15]: SPIONs loaded with a chemotherapeutic agent, e.g., mitoxantrone (MTO), are applied into the tumor supplying vascular system and are enriched in the tumor region by an external magnetic field [16]. With this, local drug concentrations can be increased, while systemic toxicity is reduced [14,16,17].

Traditionally, pharmaceutical innovations have been developed and tested in cell cultures using tumor cell lines, primary cells or mixed systems. Although two-dimensional (2D) cell culture using adherent cells is still the standard for routine high throughput screening due to easy handling, spheroidal cell culture has already proved to behave more similarly to the in vivo situation than its monolayer counterpart [18,19,20]. This includes changes in signaling and pathway activation, cell-cell-interaction, gene expression and modifications of the surrounding stroma [21,22]. These features are not observed in 2D to this extent. Therefore, multicellular tumor spheroids may serve as a more appropriate model system for avascular tumor tissue, especially for the testing of nanotherapeutics such as SPIONs.

In this study, we cultivated HT-29 colon carcinoma cells in monolayers and spheroids, to serve as model systems for uniformly and rapidly proliferating cells (2D system, artificial scenario) and cells dividing at different velocities (three-dimensional (3D) system, closer to in vivo scenario). In contrast to other previously tested cell lines, HT-29 cells were shown to form tightly packed tumor spheroids while being reproducible and easy to handle [23,24]. As the interaction with SPIONs was investigated in detail previously, MTO was the cytotoxic agent of choice in our experiments [25,26,27,28]. We compared the cytotoxic effects of both free MTO and its nanoparticle-bound form (SPION^MTO^). Depending on the cell culture system, we found completely different sensitivities towards free and SPION-bound MTO. For spheroids tested under dynamic flow conditions, the presence of a magnetic field successfully accumulated drug-loaded SPIONs.

We conclude that the type of cell culture model is crucial for the determination of realistic dose ranges for in vivo translation. SPIONs as a transporter system represent an effective delivery platform to locally increase drug concentrations, thereby possibly helping to overcome chemotherapy resistance and to increase therapeutic efficacy.

## 2. Materials and Methods

### 2.1. Synthesis of Superparamagnetic Iron Oxide Nanoparticles (SPIONs) and Loading with Mitoxantrone (MTO)

Lauric acid (LA) and human serum albumin (HSA) coated SPIONs were synthesized as described previously [28]. Briefly, after co-precipitation of iron (II) and iron (III) salts under alkaline conditions and in situ coating with LA, nanoparticles were covered by a protein corona of HSA. Physicochemical characterization of resulting SPIONs was conducted previously [28]. Sterile filtered SPIONs (0.22 µm filter) then were loaded with the chemotherapeutic drug MTO (TEVA, Pharma, Ulm, Germany) by mixing, resulting in SPION^MTO^.

The initial stock concentration of MTO was 2 mg/mL, referring to 4.5 mM. From this, 1 mL SPIONs (4.84 mg Fe/mL) was mixed freshly for every experiment with 100 µL MTO (200 µg), resulting in a SPION^MTO^ intermediate stock solution containing 450 µM MTO. For the experiments MTO and nanoparticles were diluted with H_2_O to receive the indicated final concentrations. The MTO amount in the figures is given as μM, where 0.5 μM SPION^MTO^ corresponds to 0.22 μg/mL MTO loaded on 5.38 μg/mL Fe. SPIONs were proven to be free of microbial and endotoxin contaminations before use.

### 2.2. Cells and Culture Conditions

HT-29 colon carcinoma cells (ATCC/LGC GmbH, Wesel, Germany) were cultured in McCoy’s 5A medium (Gibco^®^, Life Technologies GmbH, Darmstadt, Germany) containing 10% fetal calf serum (FCS, ThermoFisher Scientific, Waltham, MA, USA) under standard cell culture conditions in a humidified incubator (INCOmed, Memmert, Schwabach, Germany) at 37 °C, 95% humidified air and 5% CO_2_. Cells were regularly tested for mycoplasma contaminations using a polymerase chain reaction (PCR) kit Venor^®^GeM (Minerva Biolabs GmbH, Berlin, Germany). For the experiments, cells were grown to a confluence of 80–90% and passaged twice a week using 0.25% trypsin/0.02% ethylenediaminetetraacetic acid (EDTA) in phosphate buffered saline (PBS) (PANBiotech, Aidenbach, Germany). Cell count and viability were determined using MUSE^®^ Count & Viability Assay Kit in MUSE^®^ Cell Analyzer (Merck-Millipore, Billerica, MA, USA). Experiments were started when cell viability exceeded 90%.

### 2.3. Cell Culture and Treatment with SPIONS, MTO or SPION^MTO^

For monolayer culture, cells were seeded into 6-, 12- or 24-well plates (Sarstedt, Nümbrecht, Germany) in cell densities of 6, 8, 10 or 12 ⋅ 10^3^ cells per wells in 1 mL medium. Cells were incubated overnight for adherence and treated on the next day.

For spheroid culture, wells of a 96-well plate (Sarstedt, Nümbrecht, Germany) were pre-coated with 50 µL of 1.5% agarose (Roth, Karlsruhe, Germany). Then, 6, 8, 10 or 12 ⋅ 10^3^ cells were seeded into the agarose-coated wells in 100 µL of medium. The plates were shortly rotated to accumulate cells in the meniscus formed by the agarose. Cells were incubated for 72 h to form tightly packed tumor spheroids before treatment. Medium was changed every 2–3 days.

First, MTO, SPIONs and SPION^MTO^ intermediate stocks (equates to 450 µM MTO active ingredient for free MTO and SPION^MTO^ as prepared under 2.1) were diluted 1/10 with H_2_O. With this, the final test concentrations were prepared. For all experiments cells treated with the corresponding amount of H_2_O as in the samples served as negative controls.

### 2.4. Harvesting of Monolayer Cells and Spheroids

Cells grown as monolayers were washed with PBS and detached from cell culture wells with 100 µL trypsin/EDTA (PAN-Biotech GmbH, Aidenbach, Germany) for 3 min. The reaction was stopped by addition of 500 µL FCS-containing cell culture medium. All cell containing fluids were pooled in 15 mL tubes (Sarstedt, Nümbrecht, Germany) and centrifuged at 500 rcf for 5 min (Eppendorf centrifuge 5810 R, Eppendorf AG, Hamburg, Germany). The supernatants were discarded, and the cell pellet was resuspended in 120 µL PBS.

Multicellular spheroids were harvested from the 96-well plates using a cut pipette tip (1250 µL). 3–4 spheroids were pooled into 1.5 mL reaction tubes (Sarstedt, Nümbrecht, Germany), washed with PBS and single cell suspensions were prepared by incubation with trypsin for 5 min. Reaction was stopped by addition of 500 µL FCS-containing cell culture medium. Cells were centrifuged at 300 rcf for 5 min. The supernatant was discarded and cells were resuspended in 200 µL PBS.

### 2.5. Cell Proliferation

Proliferation of cells grown in monolayers was observed using IncuCyte^®^ Live-Cell Analysis system (Essen BioSciences Inc., Ann Arbor, MI, USA) for up to 100 h. Pictures were automatically taken every 1 h and the area covered by cells (confluence) was calculated and normalized to 1 for all treatment groups at the beginning of the experiment. Single cell suspensions from spheroids or adherent cells were prepared at selected time points and cells were counted with MUSE^®^ Cell Analyzer. Spheroids were monitored regularly using Axiovert 40 CFL Microscope (Zeiss, Jena, Germany) employing a 5× objective. Images of the cells were taken with Axio Vision SE64 Rel4.9 software (Zeiss, Jena, Germany) and processed in Adobe Photoshop CS6 (Adobe, San José, CA, USA). Spheroid sizes were determined with ImageJ software (National Institutes of Health, Bethesda, MD, USA). Data analysis was performed with Microsoft Excel (Microsoft, Redmond, WA, USA). Graphs and statistics were prepared using the software Prism 8 (GraphPad Software, San Diego, CA, USA).

### 2.6. Flow Cytometry

For determination of cell viability, 50 µL of single cell suspensions (two- or three-dimensional cell culture) was incubated with 150 µL of staining solution at 4 °C for 30 min under light protection. 1 mL staining solution contained 1 µL Annexin A5 conjugated with fluorescein isothiocyanate (AnnexinA5-FITC), 10 µg Hoechst 33342, (both from ThermoFisher Scientific, Waltham, MA, USA) and 66.6 ng propidium iodide (PI, Sigma-Aldrich, Hamburg, Germany) in Ringer’s solution (Fresenius Kabi Deutschland GmbH, Bad Homburg, Germany).

For determination of cell cycle and DNA degradation, 50 µL of the single cell suspensions were fixated with 400 µL of cold 70% ethanol (Carl Roth GmbH, Karlsruhe, Germany) and stored at −20 °C for further processing. The cell suspension was then centrifuged (800 rcf for 5 min), the supernatant was discarded. The cells were washed with 1 mL PBS before resuspending in 300 µL DNA extraction buffer (192 mL 0.2 M Na_2_HPO_4_ (Carl Roth GmbH, Karlsruhe, Germany) and 8 mL 0.1% Triton X-100 (Sigma-Aldrich, Hamburg, Germany), pH 7.8). Cells were incubated for 5 min at room temperature. After that, cells were centrifuged, the supernatant was removed and DNA was stained with DNA staining solution (20 µg/mL PI and 200 µg/mL ribonuclease A (Sigma-Aldrich, Hamburg, Germany) in PBS) and incubated for 30 min at room temperature in the dark. DNA content was analyzed by monitoring PI fluorescence in flow cytometry [29]. Cells were analyzed in a Gallios flow cytometer (Beckman Coulter, Fullerton, CA, USA). AnnexinA5-FITC and PI both were excited at 488 nm; the AnnexinA5-FITC fluorescence was recorded on the fluorescence (FL)1 sensor (525/38 nm band pass filter, BP) and the PI fluorescence on the FL3 sensor (620/30 nm BP). Excitation of the Hoechst 33342 fluorescence was at 405 nm and recording on the FL9 sensor (430/40 nm BP). The MTO fluorescence was monitored at 638 nm excitation and recorded on the FL7 sensor (725/20 nm BP). The measurement time was set to 120 s per tube. Depending on the treatment and cell proliferation time we recorded between 2.000 and 50.000 events per tube.

To eliminate any fluorescence bleed-through, electronic compensation was used. Data were analyzed employing Kaluza™ software Version 1.2 (Beckman Coulter, Fullerton, CA, USA) and processed in Microsoft Excel and Prism 8.

### 2.7. Magnetic Accumulation of SPION^MTO^ in Dynamic Flow Model

Ibidi µ-slides III 3D Perfusion (ibidi GmbH, Martinsried, Germany) were fully coated with 65 µL agarose per well, while maintaining a tunnel between the respective in and output channels. Cylindrical holes were created using a cut pipette tip (1250 µL), forming an artificial tumor bed. A heated needle was used to melt agarose to connect the circulation system and tumor beds. After this procedure, each well was then capable of holding four spheroids in place. Finally, five days old spheroids (seeding density 8 ⋅ 10^3^ cells) were inserted. Slides were perfused using a peristaltic pump (ISM 915, Ismatec, Wertheim, Germany) with 3 mL medium, containing SPIONs, SPION^MTO^ or soluble MTO (5 µM) which corresponds to 2.2 μg/mL MTO and/or 53.8 μg Fe/mL, respectively. A flow rate of 0.5 mL/min was maintained over a period of one hour. This setting was repeated under the influence of neodymium disc magnets (Webcraft GmbH, Gottmadingen, Germany), which were 5 mm in height and diameter and directly attached to the slide bottoms. The maximum magnetic field power measured inside the respective wells was approximately 400 mT, using Teslameter FM 302 (Projekt Elektronik Mess- und Regelungstechnik GmbH, Berlin, Germany). After the pump was stopped, spheroids were further incubated for four hours in the tumor bed. Following this, spheroids were extracted and incubated in a 96-well plate for further four days in cell culture medium containing 1% gentamycin (Biochrom, Berlin, Germany) to prevent contamination. Images were taken every day by microscopy. On day four after treatment, spheroids were analyzed by microscopy or trypsinized, stained and analyzed using flow cytometry.

## 3. Results

### 3.1. Physicochemical Characterization of SPION^MTO^

The chemotherapeutic agent MTO was freshly loaded onto SPIONs as a transporter system, resulting in SPION^MTO^ for magnetic accumulation. Previously, nanoparticles were physicochemically characterized in detail [27,28].

The hydrodynamic size of the SPIONs was characterized in water (71 ± 3.6 nm) and in Roswell Park Memorial Institute cell culture medium (RPMI-1640) (53 ± 0.8 nm). Loading of mitoxantrone increased the hydrodynamic size to 56 ± 1.0 nm in RPMI-1640. The zeta potential of SPIONs in RPMI-1640 was −11.9 ± 0.8 mV and −12.4 ± 0.5 for the non-loaded and MTO-loaded nanoparticles, respectively. The presence of albumin, iron oxides and traces of lauric acid were confirmed in the final formulation of coated SPIONs by using Fourier-transform infrared spectroscopy (FTIR). However, due to the low n/n ratio of functional groups MTO is undetectable by FTIR [25]. Freeze fracture transmission electron microscopy (TEM) showed SPIONs as multicore particles. The aggregate size determined by TEM was comparable to the size measured by dynamic light scattering (DLS) [28]. Iron oxide core size of smaller than 20 nm is a prerequisite for superparamagnetism, which is given by 10 nm diameter of every individual particle [30]. Additionally, magnetization measurements of bovine serum albumin (BSA)-coated SPIONs by SQUID indicated superparamagnetic behavior [27]. Previous binding experiments showed that 97.6 ± 0.1% of 500 µg MTO adsorbed to 1 mL of SPIONs (2 mg/mL) after 5 min equilibrium [25]. Based on these measurements, we loaded 200 µg MTO onto 4.84 mg Fe, assuming full binding of MTO to the particles. The MTO release from SPIONs in RPMI-1640 was investigated in previous studies by dialysis (11.6 ± 0.1% after 72 h) and an experimental magnetic assay setup (23.7 ± 0.4% after 72 h), indicating that MTO was released rather slowly from the nanoparticles [28]. Thus, SPION^MTO^ can be considered a drug release system with a nearly zero order kinetics, with MTO release most likely driven by diffusion from the particles according to Fick’s law.

### 3.2. Growth Kinetics of HT-29 Cells in 2D and 3D Cell Culture

**Figure 1:**
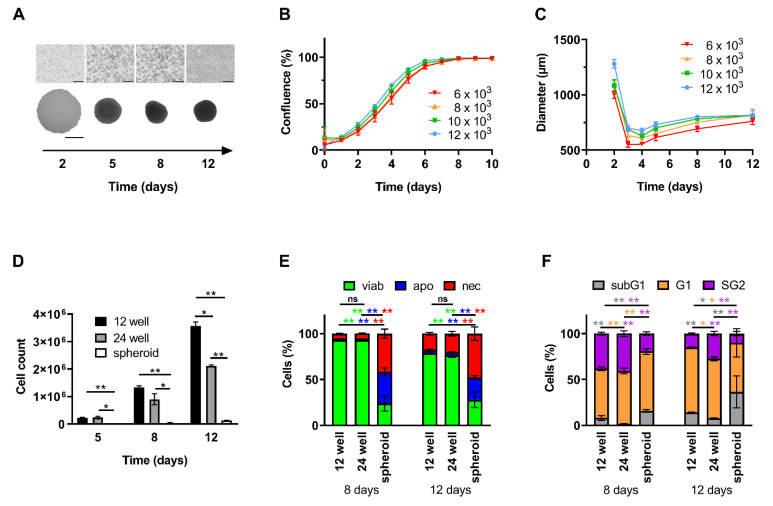
Proliferation, cell cycle and viability of cells cultured as monolayers or spheroids. HT-29 cells were seeded as monolayers (12-well plates) or spheroids (96-well plates). (**A**) Brightfield microscopy shown for 1.2 ⋅ 10^4^ cells initially seeded. Top row: monolayer cells; bottom row: spheroids, scale bars refer to 400 µm. (**B**) Confluence of monolayer cells was calculated according to brightfield pictures from IncuCyte^®^ system. (**C**) Diameters of spheroids were measured and calculated by ImageJ Software. (**D**) Absolute cell count was determined from single cell suspensions by a MUSE Cell Analyzer. (**E**) Viable, apoptotic, and necrotic cells were determined by AnnexinA5-FITC/propidium iodide (Ax/PI) staining in flow cytometry. Ax^−^PI^−^ cells are considered viable, Ax^+^PI^−^ apoptotic and PI^+^ necrotic. (**F**) Cell cycle distribution and DNA degradation were determined with propidium iodide/Triton X-100 staining showing the amount of degraded DNA (subG1 phase), diploid DNA (G1 phase), and double diploid DNA (synthesis/G2 phase). Experiments were performed 3–4 times at least in triplicates. Shown are the mean values of ≥9 wells (2D cell culture) or quadruplicates of 3 pooled spheroids (3D cell culture) with standard deviations (* *p* ≤ 0.05; ** *p* ≤ 0.01 Student’s *t*-test). Abbreviations: apo: apoptotic; Ax: Annexin A5; FITC: fluorescein isothiocyanate; MTO: mitoxantrone; nec: necrotic; viab: viable.

To compare proliferation behavior of cells growing in 2D and 3D and to reveal drug effects, cells were seeded as both monolayer and spheroids. In 2D, cells showed fast, linear and uniform proliferation as long as they were not fully confluent (Figure 1A(upper panel),B). Starting at a cell count of 1.2 ⋅ 10^4^, after 12 days, total cell counts up to approximately 2.1 ⋅ 10^6^ in 24-well plates and 3.6 ⋅ 10^6^ in 12-well plates were accomplished (Figure 1D). In contrast, spheroids contained considerably fewer cells. For a starting cell count of 1.2 ⋅ 10^4^, spheroid growth stagnated at approximately 1.3 ⋅ 10^5^ cells after 12 days of cultivation (Figure 1D). Thus, absolute cell reproduction revealed to be slower than in 2D cell culture. This observation is in line with microscopic monitoring of spheroid size: Within the first 48 h, loose cells formed large clusters. Subsequently, until day 3 spheroid size decreased and reached a diameter of 550 µm to 695 µm dependent on the amount of seeded cells, as the formation became more compact. From this point, spheroids then slowly grew and stagnated in diameter (Figure 1A,C).

Analyzing cell viabilities in 2D culture using AnnexinA5/propidium iodide (Ax/PI) staining after 12 days of incubation, viabilities were still >78.7 ± 1.3% (12-well plate) and >76.5 ± 2.3% (24-well plate), respectively (Figure 1E). In spheroids, however, cell viabilities were constantly low with 24.1 ± 8.4% and 27.9 ± 8.5% on day 8 and 12, respectively (Figure 1E). At this time, a densely packed structure was already visible within the spheroid, possibly reflecting altered cells in the center of the spheroids.

For cells seeded in 2D in 12-well plates, investigation of cell cycle and DNA content revealed a remarkable portion of 38.3 ± 1.6% (day 8) being in G2 phase, which is indicative of doubling in untreated cells, decreasing to 15.1 ± 0.8% after 12 days, indicating that proliferation stopped, probably due to full confluence of the wells. For day 12, we found a slightly increased number of cells with degraded DNA in monolayer cells, indicating cell death (Figure 1F). For spheroids, the number of cells being in G2 phase was smaller (19.4 ± 2.1% and 10.0 ± 2.3% for days 8 and 12), while the number of cells with degraded DNA was higher (15.8 ± 1.4% and 36.5 ± 17.3% for days 8 and 12) at every point of time in comparison to 2D cell culture (Figure 1F). In summary, we confirmed that HT-29 cells cultured as monolayers showed faster cell proliferation and better viability compared to cells growing in spheroids. Thus, we can consider them as model systems for uniformly or differentially proliferating tumor cells, respectively.

### 3.3. Toxicity of Free MTO Is Dependent on Cell Density and Drug Concentration

**Figure 2:**
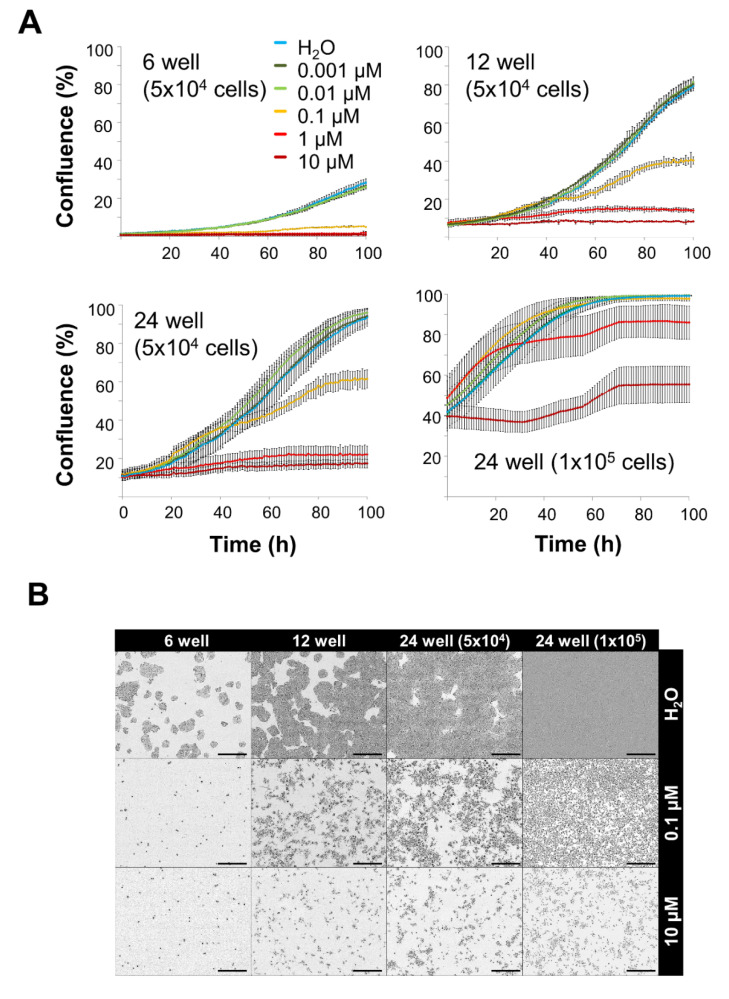
Toxicity of free mitoxantrone in monolayer cells depends on cell density. HT-29 cells were seeded into cell culture plates and treated with 0.001, 0.01, 0.1, 1 or 10 µM of free mitoxantrone (MTO). (**A**) Cell confluence of 5 ⋅ 10^4^ HT-29 cells seeded in 6-, 12-, 24-well plates and 1 ⋅ 10^5^ cells seeded in 24-well plates calculated from IncuCyte^®^ images. Shown are the mean values of 2–3 wells with 3–4 images per well with standard deviations. (**B**) Raw data files (pictures) from the IncuCyte^®^ system (t = 100 h). Scale bars refer to 400 µm. Abbreviations: MTO: mitoxantrone.

It is known that cytotoxic and cytostatic chemotherapeutics act on quickly proliferating cells [3]. Based on the proliferation kinetics of the cells (Figure 1) we speculate that cells growing in monolayers are more accessible for the toxic effects than cells growing in spheroids. Furthermore, cell density might also influence sensitivity towards toxic substances. To investigate these points, MTO was applied in various concentrations to cells grown in 6-, 12- or 24-well plates. For 24-well plates, two cell densities were tested (5 ⋅ 10^4^ cells and 1 ⋅ 10^5^ cells, respectively). Figure 2A shows cell confluence as measured by IncuCyte^®^ Live-Cell Analysis system. In 6-well plates, where cell density was low due to the large well area, MTO concentrations ≥ 0.1 µM efficiently prevented cell proliferation. In the well plates with higher cell densities per well, higher doses of MTO were required. In 12- and 24-well plates containing 5 ⋅ 10^4^ cells, ≥ 1 µM MTO had to be applied to completely stop cell proliferation. 24-well plates containing twice as much cells (1 ⋅ 10^5^) required 10 µM MTO to inhibit cell proliferation (Figure 2A,B).

Calculating the reduction of proliferation 100 h after treatment with 0.1 µM MTO revealed 81.2%, 49.2% and 34.4% for 5 ⋅ 10^4^ cells seeded in 6-, 12- or 24-well plates, respectively (Table 1). Comparing the MTO toxicities in 24-well plates containing 5 ⋅ 10^4^ or 1 ⋅ 10^5^ cells 50 h after treatment (1 µM MTO), cell proliferation was reduced by 54.0% or 9.2%, respectively. This clearly proved that cells growing in higher densities require higher drug doses (Table 2).

### 3.4. Toxicity of Free MTO Is Higher in 2D Cell Culture Compared with 3D Cell Culture

**Figure 3:**
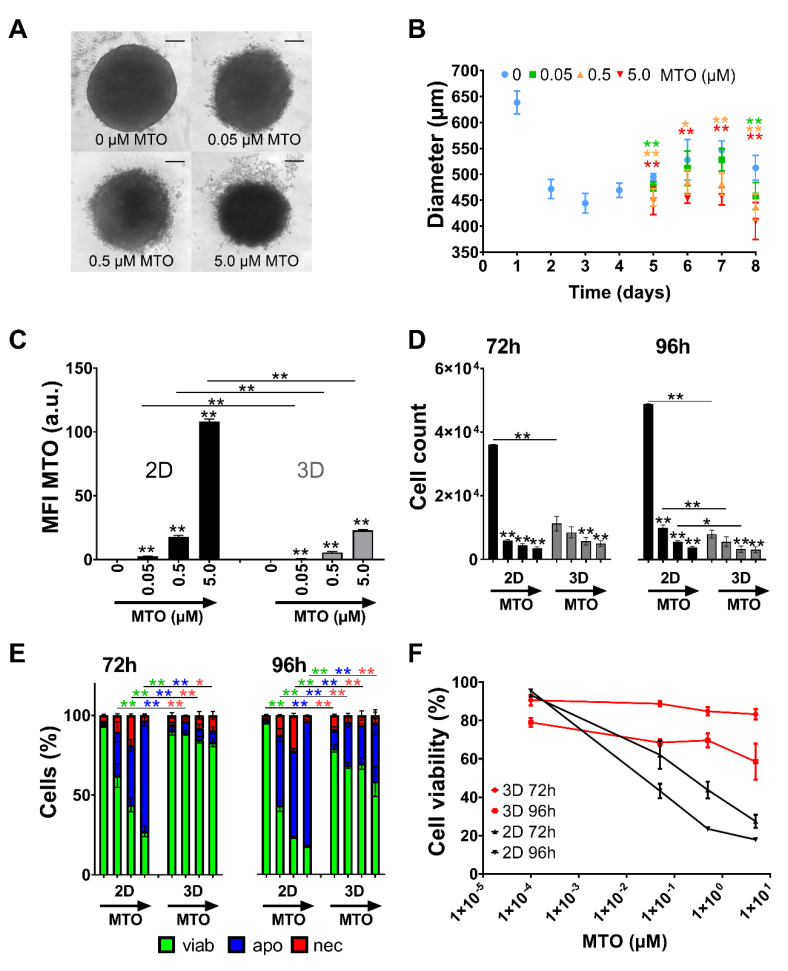
Toxicity of free mitoxantrone (MTO) depends on type of cell culture system (monolayer versus spheroid). HT-29 cells were seeded as monolayers or as spheroids and treated with various concentrations of MTO. (**A**) Brightfield microscopy of spheroids on day 4 after treatment. Scale bars refer to 100 µm. (**B**) Diameters from microscopy pictures were measured and calculated by ImageJ Software. (**C**–**E**) 0, 0.05, 0.5 and 5.0 µM MTO were used for the experiments, indicated by the arrows. (**C**) Intracellular MTO accumulation in viable cells was analyzed by mean fluorescence index (MFI, arbitrary unit, a.u.) of MTO in flow cytometry. (**D**) Relative cell count was determined from flow cytometry measurements. (**E**) Viable, apoptotic, and necrotic cells were determined by AnnexinA5-FITC/propidium iodide (Ax/PI) staining in flow cytometry. Ax^−^PI^−^ are considered viable cells (green), Ax^+^PI^−^ apoptotic (blue) and PI^+^ necrotic (red). (**F**) Non-linear fit of viable cells 72 h and 96 h (2D versus 3D cell culture). Experiments were performed in two independent experiments with triplicates (2D) or quadruplicates (3D) of 3 pooled spheroids. Shown are the mean values with standard deviations (* *p* ≤ 0.05; ** *p* ≤ 0.01 Student’s *t*-test, control versus treated samples or monolayer versus spheroid). Abbreviations: Ax: Annexin A5; FITC: fluorescein isothiocyanate; MTO: mitoxantrone; PI: propidium iodide; SPIONs: superparamagnetic iron oxide nanoparticles; SPION^MTO^: mitoxantrone-loaded superparamagnetic iron oxide nanoparticles.

Spheroids were treated with free MTO 4 days after seeding. Based on the time and dose dependency of soluble MTO (Figure 2), 0.05–5.0 µM was selected as an appropriate concentration range for further analyses. Spheroid size was analyzed on days 5 to 8 using bright-field microscopy and diameters were calculated using ImageJ software, showing that MTO induced a dose-dependent reduction in spheroid growth (Figure 3A,B). To compare toxic effects of MTO on 2D and 3D cell culture, 72 h and 96 h after treatment single-cell suspensions were prepared and analyzed in flow cytometry regarding intracellular MTO content and cell viability. Depending on cell model and applied drug dose, cells showed different amounts of intracellular MTO. Cells grown in 2D took up generally more MTO compared to cells in spheroidal culture. This applied for all tested concentrations and time points (Figure 3C). Analyzing relative cell count and viability using Ax/PI staining in flow cytometry, we found a drastic cell reduction in monolayer cell culture after treatment with MTO. 72 h after treatment, the untreated control group contained approximately 6 times more cells than the 0.05 µM MTO group. In spheroids, however, there was only a minor difference in cell number of control cells and cells treated with 0.5 µM MTO (Figure 3D). Identifying cell death phenotypes, we found a dose-dependent induction of apoptosis and secondary necrosis for monolayer cell culture after 72 h and 96 h; 72 h after treatment, untreated controls contained less than 6.5% dying/dead cells. For 0.05 µM MTO, cell death (apoptosis/necrosis) was >37.6% (>72.4% for 5 µM). In spheroids, however, after 72 h hardly any dying and dead cells were present. Cell death process started after 96 h (Figure 3E). Non-linear fitting of toxicity (reduction of Ax/PI negative cells) confirmed this finding: free MTO applied in 2D cell culture showed strong dose dependency and fast onset of toxicity. In 3D, however, cytotoxicity was not as distinct: Cell death occurred at advanced time points and was not as strong as observed in 2D (Figure 3F).

### 3.5. Comparison of SPION^MTO^ Efficacy in 2D and 3D Cell Culture

Figure 2 and Figure 3 show that MTO efficacy depended on dosage, cell density, and cell culture model (2D or 3D). Thus, MTO doses sufficient for killing tumor cells in 2D did not prevent cells from proliferating in 3D. In clinical settings, chemotherapy is often applied intravenously and is distributed not only to tumor tissue but also to other organs. Problematically, the doses of cytostatics in vivo are restricted by toxic side effects. Therefore, chemotherapeutics applied in patients are often dosed comparably low, leading to a certain need for improvement. Thus, it would be eligible to increase the toxic dose within the tumor area.

**Figure 4:**
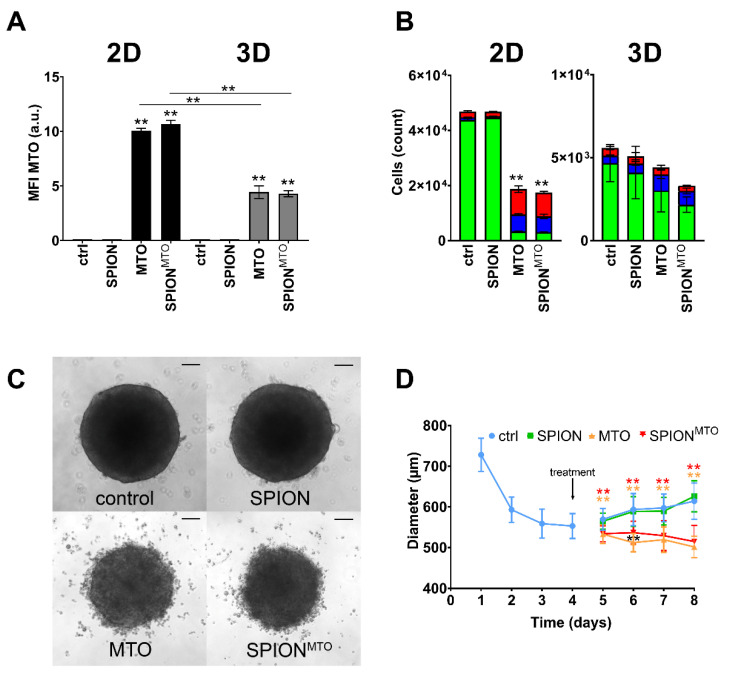
Comparison of efficacy of MTO and SPION^MTO^ in cells in monolayers or spheroids. HT-29 cells were cultivated in monolayers (2.5 ⋅ 10^4^ cells in 12-well plates) or spheroids (10^4^ cells in agarose-coated 96-well plates). After 24 h monolayer cells and after 96 h spheroids were treated with SPIONs, MTO or SPION^MTO^ (MTO content 0.5 µM each), H_2_O treated cells served as controls. For flow cytometry, single cell suspensions were prepared. Data were assessed after 96 h incubation with MTO. (**A**) Intracellular MTO accumulation in viable cells was analyzed by mean fluorescence index (arbitrary units, a.u.) of MTO in flow cytometry. (**B**) AnnexinA5-FITC/propidium iodide (Ax/PI) staining indicates viable (Ax^−^PI^−^, green), apoptotic (Ax^+^PI^−^, blue) and necrotic (PI^+^, red) cells. (**A**,**B**) Experiment was performed in two independent experiments in quadruplicates; shown are the mean values with standard deviations. (**C**) Brightfield microscopy of spheroids 4 days after treatment. Scale bars refer to 100 µm. (**D**) Sizes of spheroids were determined by ImageJ software. (**C**,**D**) The experiment was performed in three independent experiments with six to nine single spheroids per condition each. Shown are the mean values with standard deviations. Significances were calculated using Student`s *t*-test (* *p* ≤ 0.05, ** *p* ≤ 0.01; control versus treated samples, or monolayer versus spheroid; for 2B total cell counts were analysed). Abbreviations: Ax: Annexin A5; a.u. arbitrary units; FITC: fluorescein isothiocyanate; MTO: mitoxantrone; PI: propidium iodide; SPIONs: superparamagnetic iron oxide nanoparticles; SPION^MTO^: mitoxantrone-loaded superparamagnetic iron oxide nanoparticles, MFI: mean fluorescence index: MFI.

Figure 4 compares the effects of free MTO and its nanoparticle-loaded counterpart (MTO concentration 0.5 µM each). As control, the effects of the nanoparticle carrier without drug loading were investigated. Regarding intracellular MTO content, spheroids took up less MTO than cells in 2D. This observation applies for both free and nanoparticle-bound MTO, as reflected by the mean fluorescence index (MFI) for MTO and SPION^MTO^: 10.1 ± 0.2 (2D) versus 4.4 ± 0.6 (3D) and 10.7 ± 0.4 (2D) versus 4.3 ± 0.3 (3D) (Figure 4A). Concerning cell death induction, untreated cells cultivated in 2D again proliferated faster than cells in 3D. In both cases, treatment with non-loaded SPIONs did not considerably induce cell death (Figure 4B). In contrast, soluble MTO and SPION^MTO^ both induced DNA degradation and cell death strongly in 2D cell cultures and to a lesser extent in 3D cell culture. Importantly, no significant differences were detected between MTO and SPION^MTO^ in all cases (Figure 4A,B). In size monitoring via microscopy, drug-free SPIONs induced no noteworthy effect on spheroid growth (Figure 4C,D). Due to cell death induction, spheroids treated with MTO or SPION^MTO^ showed frayed outer cell layers with cells dissolved from the spheroid. Untreated spheroids, in comparison, remained tightly packed. Despite MTO and SPION^MTO^ both prevented spheroid growth, MTO was slightly more effective, with significant differences between MTO and SPION^MTO^ on day 2 after treatment (Figure 4C,D).

### 3.6. Magnetic Accumulation of SPION^MTO^ in Spheroids under Dynamic Flow Conditions

Figure 4 showed that MTO and SPION^MTO^ induced the same amount and phenotype of cell death, if applied in 2D or 3D environment, respectively. Toxic doses used in 2D cell culture, however, were not sufficient to completely inactivate cells in 3D, possibly caused by reduced drug uptake and increased cellular resistance.

**Figure 5:**
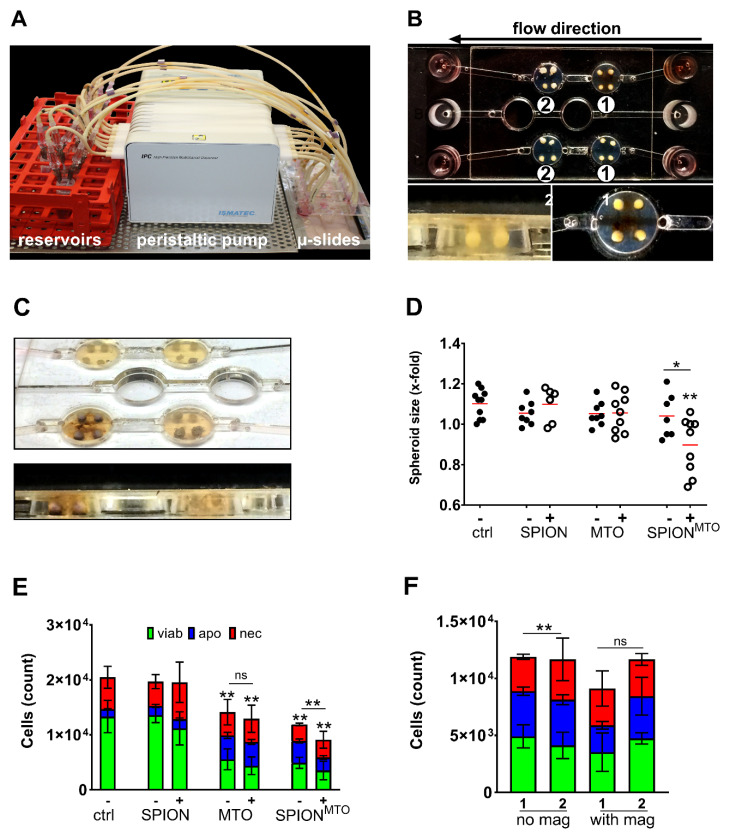
Magnetic accumulation of SPION^MTO^ in spheroids under dynamic flow conditions. (**A**) Experimental setup. A peristaltic pump transported 3 mL of medium through the Ibidi µ-slides at a constant flow rate of 0.5 mL/min. (**B**) HT-29 spheroids were added in holes pierced into the agarose coating of the flow slides. Magnets were positioned under the first wells of a row in the slides. (**C**) SPION deposits were visible around spheroids after magnetic accumulation. No change in color was observed in wells treated without magnet. (**D**) Sizes of the spheroids on day 4 after treatment with SPION, MTO or SPION^MTO^ +/− magnet. Mock treated cells served as controls. Sizes were normalized to the spheroid sizes before treatment. (**E**) AnnexinA5-FITC/propidium iodide (Ax/PI) staining of monocell suspensions prepared from spheroids on day 4 after treatment. (**F**) Comparison of cell counts (Ax/PI staining) between first and second well in serial flow. Two separated circulation systems (no magnet/with magnet) consisted of two wells in serial flow (1/2), each containing 4 spheroids exposed to SPION^MTO^. In the circulation system including a magnetic field, the magnet was positioned under only the first well (“1” in group “with magnet”). Well 2 was without magnet. Experiment was performed in two independent experiments each with four spheroids per condition. Shown are the mean values with standard deviations. Significances were calculated for total cell counts using Student’s *t*-test (* *p* ≤ 0.05, ** *p* ≤ 0.01, control versus treated samples or with versus without magnet). Abbreviations: Ax: Annexin A5; FITC: fluorescein isothiocyanate; MTO: mitoxantrone; PI: propidium iodide; SPIONs: superparamagnetic iron oxide nanoparticles; SPION^MTO^: mitoxantrone-loaded superparamagnetic iron oxide nanoparticles.

To simulate magnetically guided tumor infiltration of SPIONs, we established a dynamic flow model, containing artificial tumor beds with simplified afferent and efferent vessels. These tumor beds were designed using agarose and Ibidi µ-slides (Figure 5A,B). An artificial circulation was run by a peristaltic pump which transported the respective test compound (MTO, SPION^MTO^, SPIONs or H_2_O) through the flow slides. Each well was capable of holding four spheroids. To analyze magnetic enrichment of MTO-loaded nanoparticles in a dynamic setting, each condition (SPIONs, SPION^MTO^, soluble MTO and H_2_O) was tested twice: without and under influence of a magnet. A flow rate of 0.5 mL/min was maintained over a period of 1 h. After that, a change in color was observable in every well that was exposed to both SPIONs or SPION^MTO^ and magnetic influence, indicating accumulation of nanoparticles (Figure 5B,C). The spheroids remained in the flow slides and were incubated for further 4 h, subsequently extracted and put in 96-well plates for further 4 days. Cells were then analyzed by microscopy or flow cytometry.

Four days after treatment, spheroids exposed to MTO-free SPIONs were similar in size, cell count and cell death phenotype compared to medium control, regardless of magnetic influence. Cells exposed to pure MTO (with/without magnet) as well as SPION^MTO^ (without magnet) did not show reduction in spheroid size (Figure 5D). However, in fact, cell numbers were reduced in these conditions to the same extent (Figure 5E). SPION^MTO^ treatment in the presence of a magnet reduced not only spheroid size but also cell count compared to pure MTO or SPION^MTO^ without magnet (Figure 5D,E).

It is noteworthy that, in particular, the spheroids treated with SPION^MTO^ in the presence of a magnetic field were slightly blue in color after exposure, due to the intense accumulation of MTO. As depicted in Figure 5C, the magnetic field caused brown deposits of SPIONs in the surrounding agarose gel matrix. For these analyses only the first of the two serial wells of every slide were used.

To analyze if magnetic accumulation can remove SPION^MTO^ from circulation, spheroids from two consecutive wells on the same flow slide were analyzed (named with 1 or 2 in Figure 5B). Only the first well was under the influence of a magnetic field. Comparing spheroids treated with SPION^MTO^ in the absence of a magnetic field, all spheroids were similar in cell number and cell death phenotype. With a magnetic field affecting the first well, spheroids were different in cell number: Tumors in the first well contained only 9 ⋅ 10^3^ cells (Figure 5B number 1), tumors in the downstream well contained >1.25 ⋅ 10^4^ (Figure 5B, number 2; Figure 5F). In comparison, tumors treated with only SPION^MTO^ comprised approximately 1.2 ⋅ 10^4^ cells in both wells and showed similar cell phenotypes. For the wells treated with SPION^MTO^ under magnetic influence, we conclude that loaded nanoparticles were accumulated in the first well (resulting in reduced cell numbers). Therefore, less substance was available for the following spheroids (resulting in higher numbers).

## 4. Discussion

Treatment with chemotherapeutic agents is often accompanied by severe side effects. Accumulating cytotoxic drugs specifically in the tumor region might overcome this challenge. Dose predictions for efficient therapy from artificial cell culture systems have frequently turned out to be misleading. Here, we show that loading of a chemotherapeutic drug such as MTO onto SPIONs did not reduce its efficacy. However, this enabled us to guide the drug via magnetic forces to the desired location. Moreover, we again confirmed that dose predictions from 2D and 3D cell culture differ considerably by comparing drug effects (free and nanoparticle-bound form) using monolayer and spheroid culture. Using a peristaltic pumping system mimicking blood flow, we finally showed that SPIONs can be magnetically accumulated, locally increasing the drug concentration and drug related effects.

Two-dimensional cell culture has traditionally been used as an easy to handle routine test system for soluble drugs. Problematically, predictions for efficient therapeutic doses acquired from those systems often failed in vivo. In such systems, cells adhering on a surface are rather flat and stretched, influencing multiple processes such as proliferation, differentiation, and apoptosis [21]. Also, all cells are equally exposed to nutrients, growth factors and also applied test substances. By contrast, cells grown in 3D culture systems such as spheroids have been reported to resemble their in vivo phenotypes more closely and form a network of extracellular matrix, which might hinder the access of the drugs to be tested [31,32]. Due to differences in supply with oxygen and nutrients, the outer layers of spheroids are composed of highly proliferating cells whereas cells in the core are in quiescence or hypoxia [33]. In this study we employed HT-29 cells grown in traditional monolayer and spheroid cell culture to serve as model systems for uniformly quickly proliferating cells and those dividing in different velocities and possibly being resistant (Figure 1). Comparing different cell lines in previous experiments, HT-29 spheroids revealed to be the most suitable for our experiments due to their stability and reproducibility [23,24]. In contrast, spheroids of other cell lines were not sufficiently consistent in size or too fragile for exposure to circulation. We confirmed findings by Luca et al. [34] that HT-29 cells show different proliferation rates when cultured in 2D or 3D, respectively (Figure 1D). With prolonged incubation time, cells within our spheroids not only altered their cell cycle and went into quiescence (Figure 1F) but even cell death was induced (Figure 1E), possibly caused by lack of oxygen and nutrients. Depending on the cell culture system, we found different sensibilities towards MTO with spheroids being considerably more resistant against the toxic drug than monolayer cells (Figure 3F). Both limited drug penetration to the spheroid core (Figure 3C) and resistance of cells in hypoxic areas might explain this effect [31,35]. There is evidence that the tumor microenvironment promotes drug resistance [22,36]. Additionally, in some cancer types, upregulated expression of fibrous proteins such as collagen and fibronectin-1 was reported, indicating stroma proteins could serve as a barrier against drug diffusion [37,38,39]. As proven in the 2D cell culture (Figure 2), with dense cells being more resistant against MTO, cell density played a role regarding MTO efficacy. The impact of proliferation behavior on drug efficacy was also confirmed by studies in the past. Non-proliferating human cells showed massively reduced sensitivity for the majority of chemotherapeutics [40]. Our findings about cell density in relation to required drug concentrations (Figure 2) are supported by preceding research, as it was discussed as a major determinant contributing to reduced cytotoxic activity of certain drugs, at least for in vitro settings: This observation of reduced drug efficacy caused by increasing cell density is referred to as the inoculum effect, which is strongly pronounced with MTO [41,42].

To raise accumulation of the toxic drug in the desired area and to reduce toxic side effects, we loaded MTO onto SPIONs as magnetically guidable transporter system, which did not impair its intracellular accumulation (Figure 4A) and potency in inducing cell death (Figure 4B–D). However, the penetration of nanoparticles is discussed in terms of being dependent on size and surface functionalities, as studies in the past showed that smaller unmodified particles are more likely to penetrate deep into spheroids [43]. Goodman et. al. confirmed that particle size plays an important role, while they tend to accumulate on the spheroid surface in general. In their experiments, this limitation can be decreased to a certain degree by treatment with collagenase [44]. Also, the impact of aggregation of SPIONs on cellular uptake needs to be elaborated in the future, as our SPIONs are aggregates that consist of multiple individual nanoparticles. Existing data, however, are inconsistent in this matter. Cellular uptake of nanoparticle aggregates seems to depend on the cell type and particular endocytosis mechanisms [45,46,47]. In their work, Chithrani et al. also support the idea that different cell types require different nanoparticle properties for optimal cellular uptake [48]. Previously, we showed that our SPION^MTO^ also tend to accumulate on the outer rim of spheroids before penetrating deeper into the core, while soluble MTO penetrated faster. This was observed in terms of time dependence using life cell microscopy [24]. Based on our findings and the findings of others, we speculate that the majority of our particles (with MTO loading 56 nm in diameter) accumulate in the outer spheroid layers, at least in the beginning. It must be investigated in the future, whether MTO is released from SPIONs or remains particle-bound regarding spheroid penetration. Altogether, SPION^MTO^ reduced the growth of spheroids with the same efficacy overall (Figure 4B–D) and induced the same cell death phenotype as its soluble form. This has also been shown before by our group [23,24]. In contrast to these earlier investigations, the current spheroids revealed to be larger, which we believe is due to more frequently conducted medium changes. Also, a wide variation in size has been reported as a common weak point observed in spheroidal cell culture [21].

The magnetic targeting of SPION^MTO^ was finally proven by a newly developed flow system with spheroids placed in artificial tumor beds. Here, a peristaltic pump moved SPION^MTO^ containing cell culture medium through Ibidi µ-slides, in which spheroids have been placed into agarose beds (Figure 5A,B). In presence of magnetic fields, SPION^MTO^ were successfully accumulated in the wells, more efficiently reducing cell proliferation compared with soluble MTO or untargeted SPION^MTO^ (Figure 5D,E). This was in line with previous data on 2D cell culture, where we showed increased MTO accumulation and decreased cell proliferation in the magnetic area [49]. Additionally, the exposed spheroids on slides without influence of a magnetic field showed higher cell numbers than targeted spheroids (Figure 5F), indicating that accumulation may also reduce systemic drug availability.

It needs to be mentioned that tumor blood vessels in vivo exhibit abnormalities that are not represented in our dynamic flow model. These include irregular architecture, limited perfusion and increased permeability [50,51]. Also, endothelial cell–cell junctions form large pores, wider than those in normal endothelium [52]. These factors lead to a phenomenon of increased drug extravasation, referred to as enhanced permeability and retention (EPR) effect [52,53] and result in passive accumulation of macromolecular drugs, liposomes and nanoparticles [52]. The EPR effect therefore can support local enrichment of drugs while performing MDT with SPIONs. However, the EPR effect is not expressed to the same extent in every cancer type [54]. Individual requirements must be taken into account in order to design nanoparticles with optimal efficacy [55]. It again underlines that every tumor entity requires customized nanoparticles and that optimal physicochemical properties are crucial for adequate interaction in biomedical applications [56,57]. Nonetheless, nanoparticles tend to change over time and under different environmental conditions. This inconsistency is referred to as the chameleon effect and also affects their biological activity [58].

With our approach we showed that we can increase drug accumulation in the tumor (reflected by a spheroid). Increasing the local concentration of drugs using nanoparticles as carriers might be a useful strategy to help overcoming chemoresistance [59,60]. Also, MTO is able to induce immunogenic cell death [61]. However, this effect and the systemic immunosuppressive impact contradict each other. Reducing systemic drug availability not only could help to make use of this benefit in the future but also opens new possibilities to include other strategies, such as immunotherapy [62]. We and others previously demonstrated magnetic drug accumulation in vitro and beneficial therapeutic outcomes in vivo [15,63]. In their studies, Tietze et al. performed MDT on New Zealand white rabbits: There, conventional systemic application of soluble MTO usually led to poor drug availability in malignant tissue [16]. Less than 1% of MTO found in vivo after application was in the tumor, while most of it accumulated in the liver and spleen (up to 50%). When using SPION^MTO^ and a magnetic field instead, up to 57% of found SPION^MTO^ was in the tumor while MTO levels in the kidney and liver decreased to approximately 15%. Besides this, other groups performed MDT on mice and also found considerable increases of the carrier particles and/or the drug in the respective target areas [63,64]. On top of passive accumulation, tumor eradication could be improved by active targeting. In the past, nanoparticles have been designed with pH-responsive activity, making use of the altered pH-environment within cells with enhanced metabolism, especially cancer cells [65,66]. Since many tumors have also shown altered enzyme expression, similar effects can be achieved by nanoparticles that are enzyme-triggered and activated specifically at the tumor site [67,68]. Additionally, certain peptides can be used as coatings to bind directly to cancer cells [69,70]. Provided that tumors develop respective receptors, active targeting can also be achieved by using certain ligands [71,72]. As many strategies are capable of sparing non-malignant tissue, combining them with magnetic accumulation may be a step towards selective killing of cancer cells. This holds out the prospect of improving curative potential, while decreasing drug exposure of crucial organs.

In summary, our work shows that nanoparticles as drug transporters can raise local chemotherapeutic dose with increased induction of cell death. This applies for two-dimensional cell culture and spheroidal cell culture. Furthermore, we developed a dynamic flow system with embedded tumor spheroids to prove SPION^MTO^ accumulation and efficacy. In the future, this could help to bridge the gap between in vitro and in vivo conditions. We also think that this model system can support research on magnetically targeted nanoparticles.

## Figures and Tables

**Table 1:** Confluence of 5 ⋅ 10^4^ HT-29 cells seeded in 6-, 12-, 24-well plates as monolayers. Shown are the mean values of 2–4 wells (with 3–4 pictures per well) with standard deviations.

Cell Number	Plate Format	Well Area (cm^2^)	Confl. (%), t = 0 h	Confl. (%), t = 100 h H_2_O	Confl. (%), t =100 h 0.1 µM MTO	Residual Prol. (%)	Reduction of Prol. (%)
5⋅10^4^	6 well	9.0	1.3 ± 0.2	26.7 ± 1.6	5.0 ± 0.1	18.8	81.2
5⋅10^4^	12 well	3.5	6.3 ± 0.5	79.4 ± 1.1	40.3 ± 2.0	50.8	49.2
5⋅10^4^	24 well	1.9	11.6 ± 2.4	93.5 ± 4.7	61.4 ± 4.7	65.6	34.4

Abbreviations: confl.: confluence, prol.: proliferation.

**Table 2:** Confluence of 1 ⋅ 10^5^ cells compared with 5 ⋅ 10^4^ cells seeded in 24-well plates as monolayers. Shown are the mean values of 4 wells (with 4 pictures per well) with standard deviations.

Cell Number	Plate Format	Well Area (cm^2^)	Confl. (%), t = 0 h	Confl. (%),t = 50 h H_2_O	Confl. (%), t = 50 h 1 µM MTO	Residual Prol. (%)	Reduction of Prol. (%)
5⋅10^4^	24 well	1.9	11.6 ± 2.4	42.6 ± 7.6	19.6 ± 4.1	46.0	54.0
1⋅10^5^	24 well	1.9	38.0 ± 8.8	86.8 ± 6.1	78.6 ± 9.9	90.8	9.2

Abbreviations: confl.: confluence, prol.: proliferation.
